# Odontogenic Orbital Cellulitis at the Crossroads of Surgeries: Multidisciplinary Management and Review

**DOI:** 10.3390/diagnostics14131391

**Published:** 2024-06-29

**Authors:** Ruxandra A. Pirvulescu, Victor A. Vasile, Mihaela O. Romanitan, Mihail Zemba, Oana C. Ciulei, Aida Geamanu, Nicoleta Anton, Matei Popa Cherecheanu

**Affiliations:** 1 Department of Ophthalmology, Faculty of Medicine, Carol Davila University of Medicine and Pharmacy, 050474 Bucharest, Romania; 2 Department of Ophthalmology, Emergency University Hospital, 050098 Bucharest, Romania; 3 Department for Emergency Internal Medicine and Neurology, Stockholm South General Hospital, 11883 Stockholm, Sweden; 4 Department of Neuroradiology, Pitié-Salpêtrière University Hospital, 75013 Paris, France; 5 Department of Radiology, Clinical Emergency Hospital Bucharest, 014461 Bucharest, Romania; 6 Department of Ophthalmology, Faculty of Medicine, Grigore T Popa University of Medicine and Pharmacy, 700115 Iasi, Romania; nicolofta@gmail.com; 7 Cardiovascular Surgery Department, Carol Davila University of Medicine and Pharmacy Bucharest, 050474 Bucharest, Romania

**Keywords:** orbital cellulitis, dental complications, infection control

## Abstract

This article examines two cases of odontogenic orbital cellulitis, highlighting the complexities and interdisciplinary approaches required for effective management. We present two cases and describe the clinical challenges and treatment strategies employed. We report the diagnosis, treatment, and follow-up of patients who developed orbital cellulitis as a complication of an odontogenic infection. Our objective is to report and discuss the clinical aspects and management of this pathology compared to those observed in the literature. This study underscores the necessity for collaboration among various specialties, including ophthalmology, otolaryngology, oral surgery, radiology, and infectious disease, to address the multifaceted challenges posed by this condition. Effective management of orbital abscesses of odontogenic origin requires a timely and multidisciplinary approach for successful outcomes. This article emphasizes the importance of early diagnosis and coordinated care to prevent serious complications, such as vision loss or intracranial infections.

## 1. Introduction

Orbital cellulitis is a serious infection and inflammation affecting the soft tissues behind the orbital septum within the orbit. It is most commonly caused by the spread of an infection from the adjacent sinuses or other nearby sources. Less frequently, it can result from the bloodstream carrying the infection, the backward spread of preseptal cellulitis, or an infected eye [1]. 

Odontogenic orbital cellulitis is an inflammation of the orbit that arises from an infection associated with a tooth. It can occur due to a tooth abscess, tooth extraction, or dental surgery [2]. Orbital cellulitis can result in serious systemic complications, including meningitis, cavernous sinus thrombosis, brain abscess, and even death [1]. A common outcome of orbital cellulitis is the formation of a subperiosteal abscess, which occurs when the infection spreads beneath the periosteum of the frontal, ethmoid, or maxillary bones, leading to pus accumulation between the periorbital and orbital bones [3]. An intraorbital abscess may develop if infectious material collects within the orbit or if a subperiosteal abscess ruptures into the orbital space. Both subperiosteal and orbital abscesses can exacerbate orbital congestion, a characteristic of orbital cellulitis, and increase the risk of damage to the optic nerve, retina, and other orbital structures [1].

### 1.1. Ethyology

Orbital cellulitis develops in 70–80% of cases due to infections originating in the paranasal sinuses, especially the maxillary and ethmoidal sinuses [4]. Frequently, multiple sinuses are affected, with the ethmoid and maxillary sinuses being the most commonly co-infected [5]. It is suggested that orbital cellulitis occurs more frequently in the winter months, coinciding with a rise in sinusitis cases during this period [6]. Dental sources account for only 1.3–5% of orbital cellulitis cases [7].

The most common type of odontogenic infection is a periapical abscess, which starts when microorganisms contaminate the root canal, enter through the apical foramen, and infect the periapical tissues. The root tips of the maxillary teeth are anatomically close to nearby muscles, connective tissue, and the sinus, often leading to the development of odontogenic infections. Decay or trauma to the upper jaw teeth, as well as infected sockets following tooth extraction, can be the sources of these infections [8]. Harris has demonstrated that bacterial sinusitis develops through a sequence of events following the disruption of the normal anatomy and physiology of the sinuses [3]. Factors such as mucociliary clearance, sinus ostia openness, gas exchange, and mucosal blood flow contribute to the development of various types of infective sinusitis, including acute, nosocomial, chronic, and odontogenic sinusitis. Dental diseases can impact any of these contributing factors [9].

There are three primary pathways through which oral infections can spread to the orbit. The most common route for infection is from the oral cavity to the maxillary sinus and subsequently to the orbit, either by eroding the orbital floor bone, passing through the ethmoidal sinus, or via the infraorbital canal. The second route involves the infection spreading to the infratemporal fossa and then reaching the orbital cavity through the inferior orbital fissure. The third route is through hematogenous spread, primarily involving the facial vein as well as the superior and inferior ophthalmic veins [7,10,11].

Other causes include periocular trauma, surgical history, skin infections, dacryocystitis, oral infections, and upper respiratory infections [12]. Less common potential causes of orbital cellulitis include osteomyelitis of the orbital bones and phlebitis of the facial veins [13].

### 1.2. The Chandler Classification

The classification of orbital cellulitis dates back to 1937, when Hubert published a seminal paper detailing orbital infections originating from sinusitis [14]. In 1970, Chandler et al. updated Hubert’s system by introducing a five-stage classification, which remains in use today [15]. Known as the Chandler classification, this system categorizes the severity and location of orbital and periorbital infections. The orbital septum, a crucial anatomical landmark extending from the orbital rim to the eyelid tarsus, distinguishes between preseptal and orbital diseases.

According to this system [15],

- Stage I involves inflammation before the orbital septum, affecting the eyelids, known as preseptal cellulitis;
- Stage II is characterized by inflammation beyond the orbital septum, classified as orbital cellulitis;
- Stage III involves an infectious fluid collection between the orbital bones and their contents, termed a subperiosteal abscess;
- Stage IV is marked by an intraorbital infectious fluid collection, known as an orbital abscess;
- Stage V is characterized by phlebitis extending to the cavernous sinus, leading to bilateral eye symptoms and often severe systemic symptoms, described as cavernous sinus thrombosis.

### 1.3. Diagnosis

Clinically, orbital cellulitis is characterized by the following signs: painful exophthalmos with a sudden onset in a febrile context, eyelid edema, and chemosis. At a later stage, examination reveals limited eye movements, diplopia, and reduced visual acuity, ocular hypertension, and upon fundus examination, venous dilation, periphlebitis, and papillary edema [16]. The clinical signs of orbital infection can be ambiguous, so the initial differential diagnosis should also include noninfectious causes, including hemorrhages, tumors, allergic or inflammatory responses, and immune-mediated conditions, such as sarcoidosis and granulomatosis with polyangiitis [17].

Clinical indicators suggesting an infectious process in the orbit include a history of rhinorrhea, upper respiratory infections, sinusitis, recent orbital trauma or surgery, or immunosuppression. Symptoms such as fever, elevated white blood cell count, nasal or sinus congestion, and purulent nasal discharge can aid in confirming a diagnosis of orbital cellulitis [18]. These symptoms are not exclusively linked to oral causes, so it is crucial to investigate any dental procedures (like avulsion or endodontic treatment) conducted in the days or weeks before symptoms emerged, or any recent oral symptoms. Therefore, imaging of the facial mass is crucial when orbital cellulitis is suspected. It helps confirm the diagnosis, identify the underlying cause, and classify the type of cellulitis according to Chandler’s criteria [6].

## 2. Case 1

**Figure 1:**
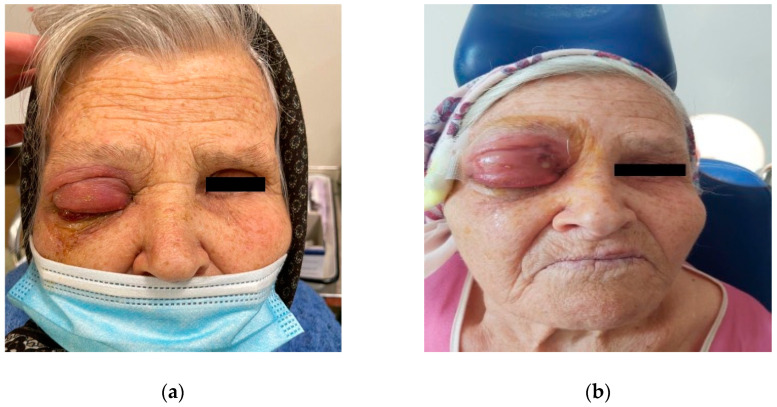
Progression of the right periorbital swelling. The condition worsened on day 3 after admission: (**a**) upon admission; (**b**) after 3 days of conservative medical treatment.

A 79-year-old woman presented to the emergency department for a 2-day history of persistent right maxillary dental pain associated with progressive eyelid swelling and marked proptosis. The patient could not open her right eye and reported no light perception when manually opening her eyelids. Clinical examination revealed complete internal and external ophthalmoplegia and axial proptosis OD with severe right erythematous periorbital and midface edema. Emphysema of the right upper eyelid was also noted (Figure 1). On presentation, her uncorrected vision was no light perception OD and 30/100 OS. The right conjunctiva was erythematous, with chemosis. The patient had a leukocytosis of 23.9, a neutrophilia of 90.3%, and biological inflammatory syndrome (CRP of 88.52 mg/L, fibrinogen of 877 mg/dL, and ESR of 78 mm/h). 

**Figure 2:**
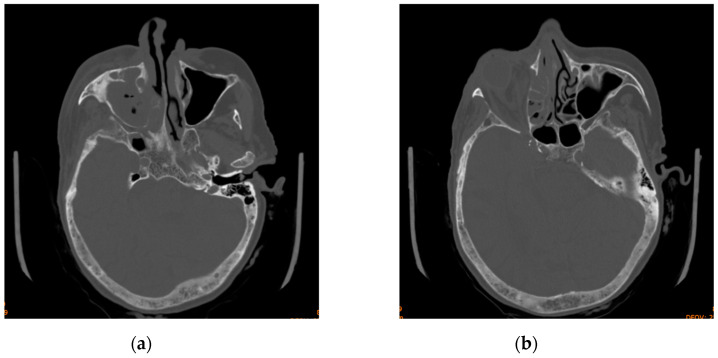
Axial section of computed tomography scan displaying (**a**) maxillary sinus opacification and (**b**) ethmoid sinus opacification.

**Figure 3:**
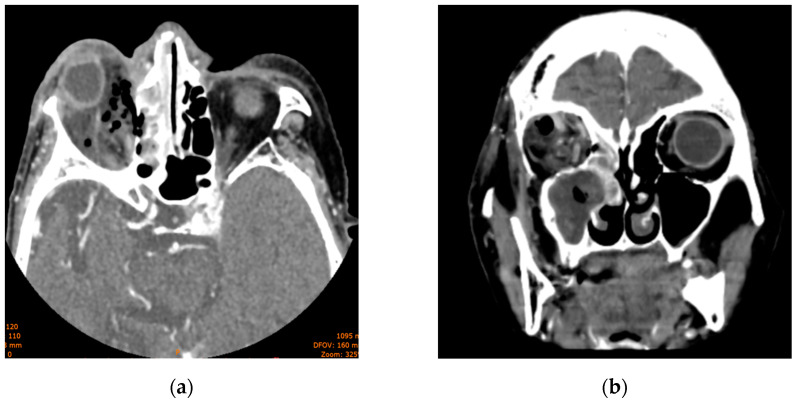
Computed tomography scan displaying maxillary and ethmoid sinus opacification, multiple gas bubbles extending from the ethmoidal cells to the right orbit, a stretched right optic nerve, and posterior globe tenting: (**a**) axial section; (**b**) coronal section.

The head CT revealed right-sided orbital abscess with reticulation of the fat, pansinusitis with maxillary and ethmoid sinus opacification (Figure 2), multiple gas bubbles extending from the ethmoidal cells to the right orbit, a stretched right optic nerve, and posterior globe tenting (Figure 3). The abscess in the right canine space originated from the buccal roots of teeth 13 and 15. The fluid collection extended continuously from the right canine space upward and backward to the right lateral orbit.

**Figure 4:**
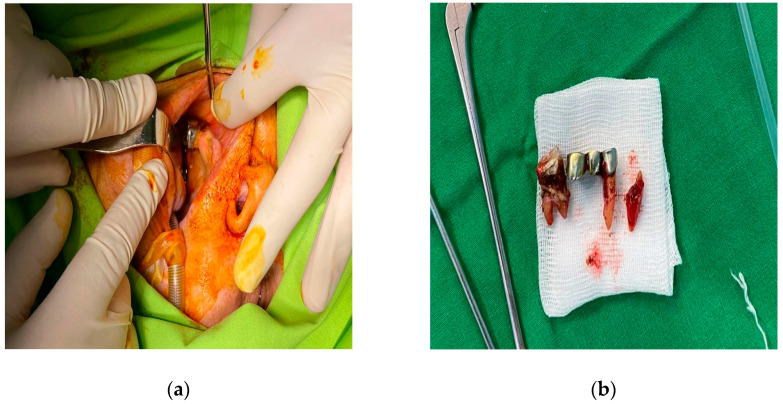
Extraction of teeth 13, 15, and 16: (**a**) intraoperative photo; (**b**) teeth 13, 15, and 16 extracted.

**Figure 5:**
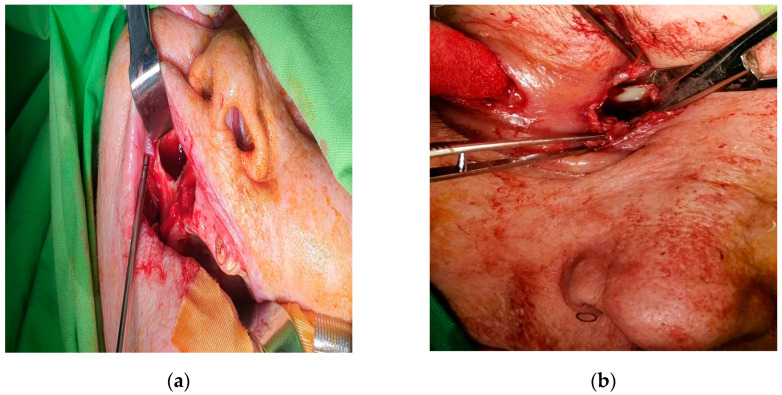
Intraoperative photo showing (**a**) maxillary drainage established intraorally via buccal vestibule; (**b**) orbital drainage via lateral canthotomy.

**Figure 6:**
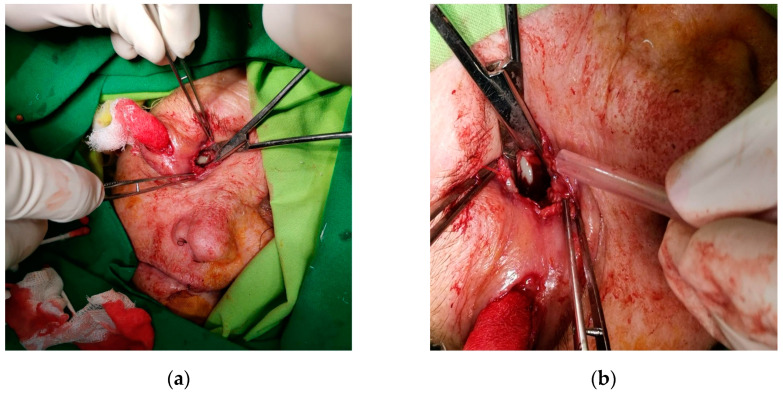
Intraoperative photo showing (**a**) orbital drainage via lateral canthotomy; (**b**) intraoperative photo depicting culture procurement through the lateral canthotomy.

**Figure 7:**
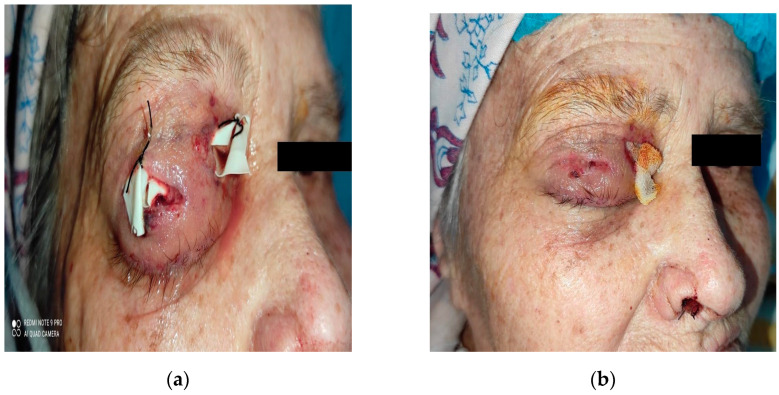
Evolution: (**a**) postoperative day 1; (**b**) postoperative day 7.

**Figure 8:**
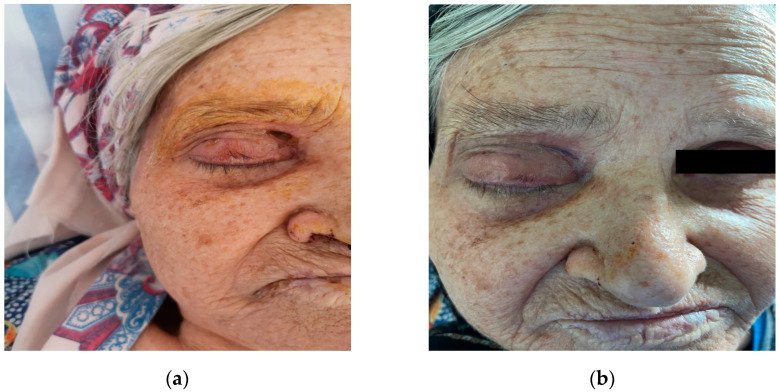
Evolution: (**a**) postoperative day 14; (**b**) postoperative month 2.

The patient was placed on empirical intravenous antibiotic coverage (metronidazole, vancomycin, and ceftriaxone). By the third day of intravenous antibiotic administration, the swelling had increased in size and progressed to generalized periorbital swelling (Figure 1). The patient underwent a right endoscopic maxillary antrostomy with the removal of maxillary sinus tissue, followed by a right endoscopic total ethmoidectomy. Additionally, surgical drainage of multiple loculated intraorbital abscesses and excision of necrotic orbital fat were performed through a sub-brow incision, along with the extraction of teeth 13, 15, and 16 (Figure 4, Figure 5 and Figure 6). The anesthesia service provided general anesthesia via an oral endotracheal tube. The patient’s recovery after surgery was uneventful. The swelling diminished quickly on the first day after the surgery (Figure 7). The Penrose drain was removed on postoperative day 3. Before discharge, clinical improvement was noted with a decrease in right periorbital swelling (Figure 8). On follow-up 2 months later, visual acuity was unchanged.

## 3. Case 2

**Figure 9:**
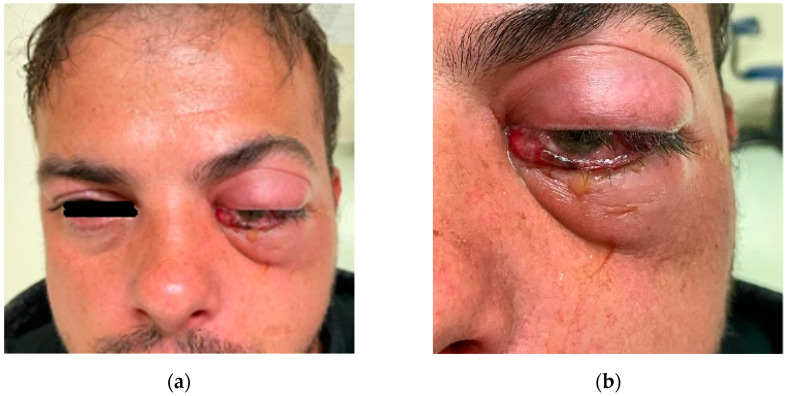
Clinical aspect on presentation: (**a**) face aspect; (**b**) OS aspect.

A 35-year-old man presented to the emergency department for a 2-day history of persistent left maxillary dental pain associated with progressive eyelid swelling. The patient received dental treatment (root canal treatment) two days before. One day after the root canal treatment, the patient experienced left eye pain with eyelid swelling. Oral antibiotic therapy (Augmentin 1 g twice a day) was prescribed by the dentist, and the patient was sent home. On the next day, the patient’s condition deteriorated, and he was transferred by ambulance to the emergency department of the University Emergency Hospital in Bucharest. On presentation, a clinical examination revealed painful axial proptosis of the left eye, and complete internal and external ophthalmoplegia (Video S1) with severe left erythematous periorbital and midface edema (Figure 9). The left conjunctiva was erythematous, with chemosis. On presentation, his uncorrected vision was counting fingers OS. The patient had a leukocytosis of 13.1, a neutrophilia of 9.9, and biological inflammatory syndrome (CRP of 61.8 mg/L, fibrinogen of 608 mg/dL, and ESR of 44 mm/h).

**Figure 10:**
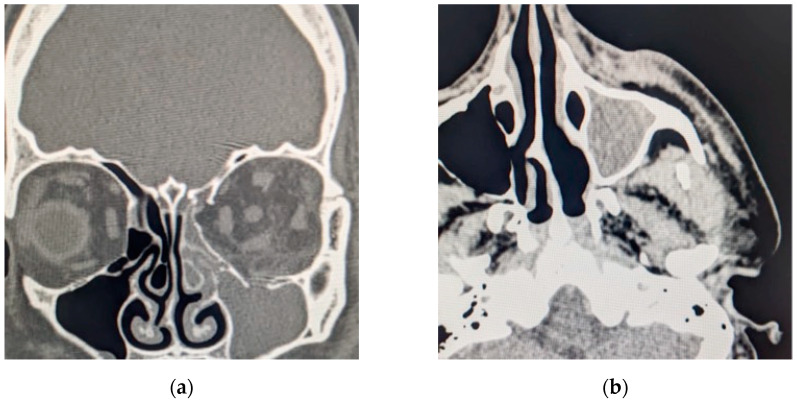
Computed tomography scan section displaying (**a**) coronal section with maxillary sinus opacification and orbital abscess; (**b**) axial section with maxillary sinus opacification.

The head CT revealed a large left-sided orbital abscess of 25 × 14 mm, a left preseptal eyelid abscess with a thickness of 9 mm, left maxillary sinus opacification, posterior globe tenting, and retromaxillary soft tissue swelling with a thickness of 7 mm with extension in the orbit via the inferior orbital (Figure 10).

**Figure 11:**
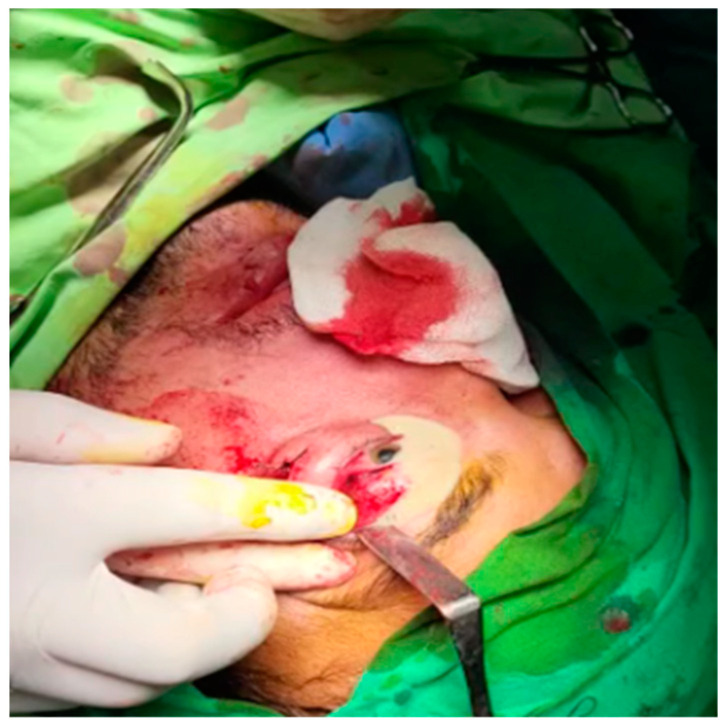
Intraoperative photo following access in the orbit; note the externalization of a large amount of purulent discharge.

**Figure 12:**
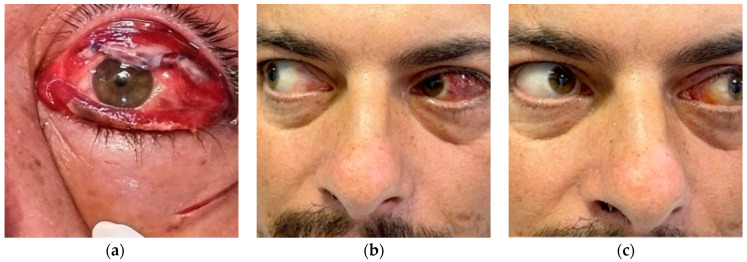
Evolution: next day after surgery (**a**), 2 weeks after surgery (**b**), and 2 months after surgery (**c**).

The patient was placed on empirical intravenous antibiotic coverage (metronidazole and ceftriaxone). He underwent left endoscopic maxillary antrostomy with removal of maxillary sinus tissue, left endoscopic ethmoidectomy, and then surgical orbital drainage (Video S2) via a superior transconjunctival approach 3 mm from the upper limbus (Figure 11). The anesthesia service provided general anesthesia via an oral endotracheal tube. The patient’s recovery after surgery was uneventful. The swelling diminished quickly on the first day after the surgery. Before discharge, clinical improvement was noted, with a decrease in left periorbital swelling (Figure 12). Best corrected visual acuity (BCVA) started to improve, becoming 20/20 on follow-up 2 months later.

## 4. Discussion

Patients exhibiting visual symptoms or significant periorbital swelling with restricted extraocular movement should be promptly assessed by a specialist, and CT imaging is recommended [19]. The initial management of odontogenic orbital infections involves empirical antibiotic therapy targeting aerobic gram-positive and anaerobic bacteria, as well as common oral pathogens [20,21].

Over time, the medical and surgical treatments for orbital cellulitis have advanced significantly. In the early twentieth century, treatment options were limited. However, since antibiotics were introduced for managing orbital cellulitis in the 1940s, there has been a decline in the incidence of complications associated with the condition [1]. Medical treatment involves the prompt start of broad-spectrum intravenous antibiotics. The most frequently reported treatment in the literature is a combination of metronidazole with a third-generation cephalosporin, although other combinations have also proven effective. This regimen targets both aerobic and anaerobic organisms, with anaerobes often playing a significant role in odontogenic orbital cellulitis [7].

The rollout of the *H. influenzae* vaccine has significantly reduced the prevalence of *H. influenzae* as the leading cause of orbital cellulitis and has also decreased the overall incidence of orbital infections in children [22,23]. In recent years, the rising antibiotic resistance among bacteria has become a major concern, particularly with the increasing prevalence of community-acquired methicillin-resistant Staphylococcus aureus. Approximately 20% of *S. aureus* strains from orbital and sinus cultures have been identified as MRSA [24]. Furthermore, a study in France found that 30% of *H. influenzae* and 80% of Moraxella strains are resistant to beta-lactam antibiotics [25]. To combat the increasing problem of antibiotic resistance, the Centers for Disease Control and Prevention and the World Health Organization have proposed several strategies to slow its progression [26]. The guidelines recommend prescribing antibiotics only when necessary and selecting them based on the identification of the causative pathogens and their drug susceptibility. They also advise transitioning to oral therapy when appropriate and emphasize the importance of completing the full course of antibiotic treatment [27].

Recent studies have demonstrated that using corticosteroids to treat orbital cellulitis with subperiosteal abscess can reduce the incidence of adhesions, sinus swelling, and stenosis. When used alongside systemic antibiotics, corticosteroids enhance perioperative surgical results thanks to their anti-inflammatory properties [28,29]. Neelam Pushker evaluated the effectiveness of corticosteroid therapy in cases of acute sinusitis and orbital cellulitis by comparing the outcomes of patients treated with intravenous antibiotics alone to those receiving a combination of intravenous antibiotics and systemic corticosteroids for orbital cellulitis [29]. Administering high-dose intravenous corticosteroids led to a quicker alleviation of symptoms, including fever, pain, periorbital edema, exophthalmos, and restricted eye movement. Additionally, these corticosteroids facilitated a faster improvement in visual acuity, although they did not affect the long-term recovery of visual acuity. The group that received corticosteroid therapy also experienced a significantly shorter hospital stay [7].

Systemic antibiotics continue to be the first line of treatment for orbital cellulitis, with surgical drainage being employed for cases involving abscess formation, visual impairment, or lack of response to antibiotic therapy [30]. When radiographic data show an orbital abscess, or there is initial poor vision, or an escalation of orbital signs and/or deteriorating vision despite systemic antibiotics, it is recommended to drain the orbital abscess and the affected sinuses [31]. The procedure generally involves routine orbital drainage, often combined with maxillary sinus drainage in cases of cellulitis corresponding to Chandler stages III and IV. The specific approach is guided by prior imaging results.

The Garcia–Harris criteria, which take into account the patient’s age at presentation and the infection’s location, are used to decide whether surgical intervention is appropriate [13]. Nonsurgical management with close monitoring and intravenous antibiotics is recommended for patients with a subperiosteal orbital abscess only under specific conditions: the patient must be younger than 9 years old, there should be no involvement of the brain or frontal sinus, no dental abscess, no vision loss or afferent pupillary defect, and the abscess on the medial wall should be moderate in size or smaller [32,33].

Recent advancements in medical technology, particularly the adoption of transnasal endoscopic sinus surgery, have significantly reduced the need for external incisions to drain sinuses. Procedures such as the Caldwell–Luc operation have become outdated, and external ethmoidectomy is now generally reserved for cases where endoscopic visibility is inadequate or when orbital symptoms do not improve [34,35]. Transnasal endoscopy is widely recognized as a safe and effective approach for managing subperiosteal abscesses [36]. It minimizes scarring, accelerates the reduction in periorbital swelling, and decreases the risks of bleeding and further infection spread by avoiding periorbital incisions [34]. It is crucial to address and eliminate the source of the oral infection, which often involves extracting the problematic tooth and performing oral drainage if required [7]. 

One of the limitations of our study concerns the absence of identification in culture of the germ responsible for the infection, as our patients were already under broad-spectrum antibiotic therapy when the sample was taken since, on presentation, their severe condition required urgent empirical antibiotic therapy. Additional research would be necessary to adapt prophylactic antibiotic therapy to local specificities in the case of dental treatments.

## 5. Conclusions

Orbital cellulitis resulting from an odontogenic cause is a rare but serious complication that can lead to permanent vision loss. Early diagnosis of this condition is crucial for providing timely treatment and preventing severe outcomes, including total blindness or life-threatening consequences. The prognosis for visual recovery is generally poor if significant vision loss is present at the time of diagnosis. Practitioners should be aware of this uncommon cause of orbital cellulitis, as it may require intensive monitoring, serial imaging, multidisciplinary care, and surgical intervention.

## Data Availability

The original contributions presented in this study are included in the article/Supplementary Materials; further inquiries can be directed to the corresponding author/s.

## Supplementary Materials

The following supporting information can be downloaded at https://www.mdpi.com/article/10.3390/diagnostics14131391/s1: Video S1: Ophthalmoplegia; Video S2: Surgical drainage.

## References

[1] Yen M.T., Johnson T.E. (2017). Orbital Cellulitis and Periorbital Infections.

[2] Youssef O.H., Stefanyszyn M.A., Bilyk J.R. (2008). Odontogenic Orbital Cellulitis. Ophthalmic Plast. Reconstr. Surg..

[3] Harris G.J. (1993). Age as a Factor in the Bacteriology and Response to Treatment of Subperiosteal Abscess of the Orbit. Trans. Am. Ophthalmol. Soc..

[4] Procacci P., Zangani A., Rossetto A., Rizzini A., Zanette G., Albanese M. (2017). Odontogenic Orbital Abscess: A Case Report and Review of Literature. Oral Maxillofac. Surg..

[5] El-Sayed Y. (1995). Orbital Involvement in Sinonasal Disease. Saudi J. Ophthalmol..

[6] Nageswaran S., Woods C.R., Benjamin Jr D.K., Givner L.B., Shetty A.K. (2006). Orbital Cellulitis in Children. Pediatr. Infect. Dis. J..

[7] Guichaoua C., Genest-Beucher S., Boisrame S. (2024). Odontogenic Orbital Cellulitis: Literature Review. J. Oral Med. Oral Surg..

[8] Sharma A., Ingole S., Deshpande M., Ranadive P., Sharma S., Chavan A. (2021). An Insight on Management of Odontogenic Orbital Infections: Report of Two Cases. J. Oral Med. Oral Surg..

[9] Mehra P., CAIAZZO A., BESTGEN S. (1999). Odontogenic Sinusitis Causing Orbital Cellulitis. J. Am. Dent. Assoc..

[10] Muñoz-Guerra M.F., González-García R., Capote A.L., Escorial V., Gías L.N. (2006). Subperiosteal Abscess of the Orbit: An Unusual Complication of the Third Molar Surgery. Oral Surg. Oral Med. Oral Pathol. Oral Radiol. Endod..

[11] Poon T.L., Lee W.Y., Ho W.S., Pang K.Y., Wong C.K. (2001). Odontogenic Subperiosteal Abscess of Orbit: A Case Report. J. Clin. Neurosci..

[12] de Medeiros E.H.P., Pepato A.O., Sverzut C.E., Trivellato A.E. (2012). Orbital Abscess during Endodontic Treatment: A Case Report. J. Endod..

[13] Harris G.J. (1983). Subperiosteal Abscess of the Orbit. Arch. Ophthalmol..

[14] Hubert L. (1937). Orbital Infections Due to Nasal Sinusitis. NY State J. Med..

[15] Chandler J.R., Langenbrunner D.J., Stevens E.R. (1970). The Pathogenesis of Orbital Complications in Acute Sinusitis. Laryngoscope.

[16] Hamedani M., Ameline-audelan V., Morax S. (2002). Affections Inflammatoires de l’orbite. Encycl. Med. Chir. (Ed. Sci. Médicale Elsevier) Opthalmologie.

[17] Hong E.S., Allen R.C. (2010). Orbital Cellulitis in a Child. https://webeye.ophth.uiowa.edu/eyeforum/cases/103-Pediatric-Orbital-Cellulitis.htm.

[18] Meara D.J. (2012). Sinonasal Disease and Orbital Cellulitis in Children. Oral Maxillofac. Surg. Clin..

[19] Houle A.N., Pham C., Valikodath N., Elmowitz J.S., Callahan N. (2021). Odontogenic Subperiosteal Abscess of the Lateral Orbit: Timely Recognition and Management. Eur. J. Dent..

[20] STüBINGER S., Leiggener C., Sader R., Kunz C. (2005). Intraorbital Abscess: A Rare Complication after Maxillary Molar Extraction. J. Am. Dent. Assoc..

[21] Stefanopoulos P.K., Kolokotronis A.E. (2004). The Clinical Significance of Anaerobic Bacteria in Acute Orofacial Odontogenic Infections. Oral Surg. Oral Med. Oral Pathol. Oral Radiol. Endod..

[22] Thakar A., Tandon D.A., Thakar M.D., Nivsarkar S. (2000). Orbital Cellulitis Revisited. Indian J. Otolaryngol. Head Neck Surg..

[23] Shaikh M.R., Baqir S.M., Zakir N. (2004). Orbital Cellulitis Masquerading as Cavernous Sinus Thrombosis: A Case Report. J. Pak. Med. Assoc..

[24] Bedwell J., Bauman N.M. (2011). Management of Pediatric Orbital Cellulitis and Abscess. Curr. Opin. Otolaryngol. Head Neck Surg..

[25] Gehanno P., Beauvillain C., Bobin S., Chobaut J.-C., Desaulty A., Dubreuil C., Klossek J.-M., Pessey J.-J., Peyramond D., Strunski A. (2000). Short Therapy with Amoxicillin-Clavulanate and Corticosteroids in Acute Sinusitis: Results of a Multicentre Study in Adults. Scand. J. Infect. Dis..

[26] Bertino Jr J.S. (2009). Impact of Antibiotic Resistance in the Management of Ocular Infections: The Role of Current and Future Antibiotics. Clin. Ophthalmol..

[27] Seltz L.B., Smith J., Durairaj V.D., Enzenauer R., Todd J. (2011). Microbiology and Antibiotic Management of Orbital Cellulitis. Pediatrics.

[28] Ramadan H.H. (2001). Corticosteroid Therapy during Endoscopic Sinus Surgery in Children: Is There a Need for a Second Look?. Arch. Otolaryngol. Head Neck Surg..

[29] Pushker N., Tejwani L.K., Bajaj M.S., Khurana S., Velpandian T., Chandra M. (2013). Role of Oral Corticosteroids in Orbital Cellulitis. Am. J. Ophthalmol..

[30] Jain A., Rubin P.A.D. (2001). Orbital Cellulitis in Children. Int. Ophthalmol. Clin..

[31] Henry C.H., Hughes C.V., Larned D.C. (1992). Odontogenic Infection of the Orbit: Report of a Case. J. Oral Maxillofac. Surg..

[32] Smith J.M., Bratton E.M., DeWitt P., Davies B.W., Hink E.M., Durairaj V.D. (2014). Predicting the Need for Surgical Intervention in Pediatric Orbital Cellulitis. Am. J. Ophthalmol..

[33] Garcia G.H., Harris G.J. (2000). Criteria for Nonsurgical Management of Subperiosteal Abscess of the Orbit: Analysis of Outcomes 1988–1998. Ophthalmology.

[34] Manning S.C. (1993). Endoscopic Management of Medial Subperiosteal Orbital Abscess. Arch. Otolaryngol. Head Neck Surg..

[35] Wolf G., Anderhuber W., Kuhn F. (1993). Development of the Paranasal Sinuses in Children: Implications for Paranasal Sinus Surgery. Ann. Otol. Rhinol. Laryngol..

[36] Rahbar R., Robson C.D., Petersen R.A., DiCanzio J., Rosbe K.W., McGill T.J., Healy G.B. (2001). Management of Orbital Subperiosteal Abscess in Children. Arch. Otolaryngol. Head Neck Surg..

